# Changes in Serum Protein Profile in Laying Hens Housed in a Cage-Free System

**DOI:** 10.1155/vmi/4135744

**Published:** 2024-12-07

**Authors:** Csilla Tóthová, Edina Sesztáková, Blanka Galiková, Veronika Glembová, Veronika Oršuľaková, Oskar Nagy

**Affiliations:** ^1^Clinic of Ruminants, University of Veterinary Medicine and Pharmacy in Košice, Komenského 73, Košice 041 81, Slovakia; ^2^Clinic of Birds, Exotic and Free Living Animals, University of Veterinary Medicine and Pharmacy in Košice, Komenského 73, Košice 041 81, Slovakia

## Abstract

The objective of this study was to characterize the distribution of serum proteins in laying hens reared under cage-free open farm conditions and to evaluate the changes in the concentrations of serum protein fractions throughout the 1-year laying period. Ten Dekalb White white-egg–laying hens were blood sampled from the wing vein at 17, 22, 29, 38, 50, 60, and 70 weeks of age. Egg laying was observed at 18 weeks of age. The average daily egg production in the initial phase of laying (week 22) was 6.3, reaching the maximum (8.5 eggs) in week 38. After a slight decrease, from week 60, the egg production increased repeatedly and stayed relatively constant till the end of the study (8.1–8.3 eggs). The serum samples were analyzed for the concentrations of total proteins and the electrophoretic pattern of serum proteins. Five protein fractions were identified on the serum protein electrophoretogram of the hens, including albumin, *α*_1_-, *α*_2_-, *β*-, and *γ*-globulins. The size and shape of the fractions differed significantly according to the stage of the laying period. Significant changes were detected in the ratio of all individual protein fractions as well as in the concentrations of total proteins and protein fractions. The concentrations of total proteins, albumin, *α*_2_-, *β*-, and *γ*-globulins decreased significantly after the beginning of egg production compared to values recorded before laying (*p* < 0.05) and started to increase after reaching the maximum egg production in week 38. The A/G ratios were higher before and at the beginning of the laying period, and the lowest values were obtained in weeks with the highest egg production. These findings suggest that the beginning of egg production is the most critical period in the laying cycle of hens reared under alternative farming conditions characterized by the most marked alterations in the serum protein profile.

## 1. Introduction

Proteins are substantial compounds of the blood serum or plasma with many different physiological roles in the body and are essential for the maintenance of homeostasis. Furthermore, they serve as an important production indicator in some animal species [1]. Serum proteins exist at a constant ratio under normal physiological conditions, but there are many biological and physiological factors that may affect their standard electrophoretic tracing [2]. From these, states of stress, the biological rhythm specific to each animal, age, feeding, molting, and nest-building are the most important factors that may potentially affect the concentrations of serum proteins also in birds [3]. The high potential of egg production and laying performance of actual laying hen breeds place enormous demands on their metabolism. Therefore, laying and intensive egg production in hens may be accompanied by major physiological and biochemical alterations, especially by changes in blood parameters related to protein metabolism.

Electrophoresis of serum proteins is considered to be one of the most widely used diagnostic techniques for the evaluation of alterations in the protein profile and standard fractionation of blood proteins in clinical biochemistry [4]. It has also been studied in animal medicine, in particular for the diagnosis of diseases characterized by dysproteinemia or to identify the type of inflammation and stimulated humoral immune response [5]. Serum protein electrophoresis has been successfully introduced also to avian medicine, where it is mainly used for the diagnosis of inflammatory processes associated with bacterial, viral, or parasitic infections [6]. Seeing that there are many anatomic and functional differences between birds and mammals, difficulties may arise in the identification of different protein fractions that do not migrate to the same zone compared to mammalian species [7, 8]. Furthermore, the number of separated fractions and the general appearance of the electrophoretic curve in avian species are different compared to mammals [9, 10].

In hens, several studies have been conducted to describe intensive metabolic processes and biochemical changes associated with high egg production during the laying period. However, the majority of existing data regarding the changes in biochemical parameters during the ovulatory cycle are related to the mineral profile and bone metabolism [11]. They are based on investigations made decades ago predominantly in hens from commercial housing systems. Some studies were carried out also to determine the serum proteins and their changes in hens during the laying cycle [12–14]. However, these studies were focused especially on the examination of total serum protein concentrations or albumin throughout the laying cycle. Regarding the serum protein fractions and their distribution in laying hens, there are scarce up-to-date data. Therefore, in order to obtain new knowledge, the present study aimed to describe the electrophoretic pattern of blood serum proteins, as well as to evaluate the changes in the concentrations of protein fractions in laying hens reared under open farm conditions during the 1-year egg-laying period, including the prelaying, laying, and the end of the laying cycle.

## 2. Materials and Methods

### 2.1. Ethical Approval

This study was performed in accordance with the ethical standards and guidelines approved by the Institutional Committee on protection of animals used for scientific purposes and complied with the institutional requirements of the Code of Ethics for Scientists (under Directive 74/2019/UVLF). The blood samples from the hens were collected as per standard sampling procedure used without any harm to the animals.

### 2.2. Animals and Sample Collection

This study was performed on a small local poultry farm under conventional free-range cage-free farming conditions between March 2022 and April 2023. Ten Dekalb White white-egg–laying hens at the age of 14 weeks were included in the evaluation. The hens were housed in sheds overnight, which were opened during daylight hours to the outdoor area, and the hens had access to pasture. In the open breeding area, the hens also had a shelter in case of the possibility of protection from sunlight in the summer or from rain in case of adverse weather. The lighting was natural according to the actual season of the year. The length of the light day was not extended by artificial lighting. The laying nests were placed in the building, where the hens were located at night. Eggs were collected once a day in the afternoon. The initial body weight of the hens was 1.50 ± 0.15 kg on average, which increased to 1.74 ± 0.20 kg in week 29 and then remained relatively constant up to the end of the evaluated 1-year laying cycle. In the prelaying period, the hens were fed with a commercial starter feed (GCH) with wheat grain and from week 17 with the grower feed Chicken Midi (LCH). Subsequently from week 20, the layer hens classic (LHC) and feed for commercial laying hens (LHCo) mixed in a ratio of 1 to 1 with wheat grain until the end of the evaluated laying period was fed (Energys Hobby, DeHeus, Czech Republic). The main chemical composition of the diets and additives in the diet are presented in Table 1. Feed was replenished for laying hens during the day to ensure the possibility of its continuous intake according to their needs. Feed was provided *ad libitum* with free access to water. The health status of the animals was evaluated weekly until the end of the study using routine diagnostic procedures and was oriented to the observation of general health state, feed intake, egg production, and behavior. The evaluated hens were in good general health without any obvious clinical signs of diseases. Egg laying was observed at 18 weeks of age. The average daily egg production in the initial phase of laying (week 22) was 6.3 and then increased gradually, reaching the maximum (8.5 eggs) in week 38. After a slight decrease (7.8 eggs on average) in week 50, from week 60, the egg production increased repeatedly and stayed relatively constant till the end of the study (8.1–8.3 eggs).

Blood samples were obtained in several periods of the laying cycle. First, sampling was performed before the onset of the egg laying in the 17th week of age. Further blood collection was made after the onset of lay in the 22nd and 29th weeks, in week 38 close to the peak of the laying period, during laying persistency at 50 and 60 weeks, and at the end of the evaluated laying period in week 70. About 1 mL of blood was collected from the wing vein into serum gel separator tubes without additives and anticoagulants (Sarstedt, Nümbrecht, Germany). Serum was separated after letting blood samples to coagulate at room temperature and centrifugation at 4000 g for 15 min. The serum was separated from the clot and dispensed into plastic tubes. No hemolysis was present in the separated sera. The serum samples were stored in a freezer at −20°C and analyzed within 1 month.

### 2.3. Laboratory Analyses

The serum samples were analyzed for the concentrations of total serum proteins (TP, g/L) and the electrophoretic pattern of serum proteins. The biuret method was applied to measure the TP concentrations using commercially available diagnostic kits (Randox, Crumlin, United Kingdom) and the automated chemistry analyzer Alizé (Lisabio, Pouilly en Auxois, France). The separation and distribution of serum protein fractions was performed by zone electrophoresis on agarose gel using an automated electrophoresis system Hydrasys with commercial diagnostic kits Hydragel 7 Proteine (Sebia Corporate, Lisses, Evry Cedex, France) according to the application instructions of the manufacturer. The analyses of serum protein fractions were performed according to the methods presented by Tóthová et al. [15]. The protein fractions were expressed as relative values (%) according to the optical density, and their absolute concentrations (g/L) were quantified from the TP concentrations. Albumin:globulin ratios (A/G) were calculated as well.

### 2.4. Statistical Analysis

Mean values and standard deviations were calculated as descriptive statistics using the computer program GraphPad Prism V5.02 (GraphPad Software Inc., California, USA). The Kolmogorov–Smirnov test for normality was applied to evaluate the distribution of the data. Data were submitted for analysis of variance using the Kruskal–Wallis test. The significance of differences among the various sample collections during the laying period was assessed using Dunn's test. Differences were considered significant when the *p* value was less than 0.05.

## 3. Results

**Figure 1:**
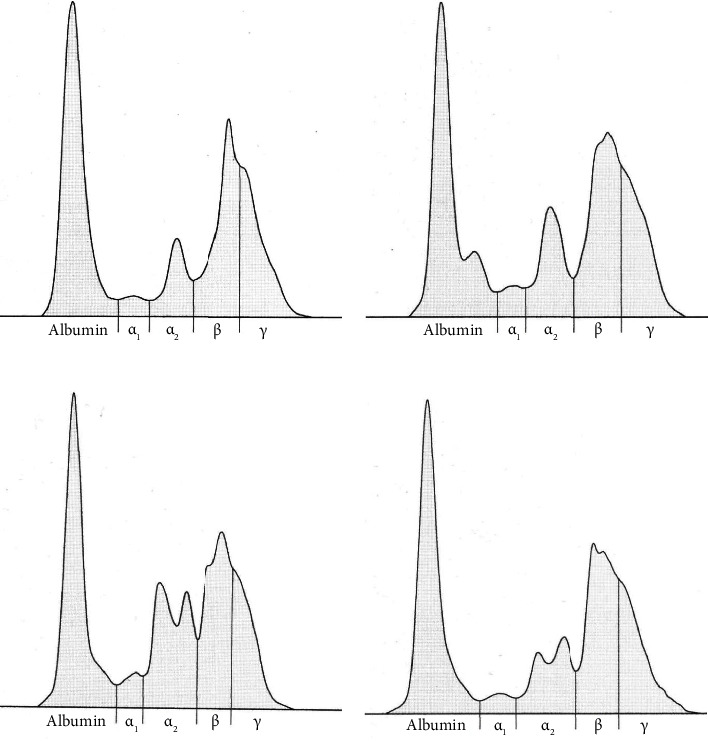
Representative electrophoretograms characterizing the changes in serum protein fractions in laying hens before start of laying (a) and during laying at the age of 22 (b), 38 (c), and 60 weeks (d).

Five protein fractions were identified on the serum protein electrophoretogram of the hens, including albumin, *α*_1_-, *α*_2_-, *β*-, and *γ*-globulins, but the size and shape of separated serum protein fractions differed according to the stage of the laying period (Figures 1(a), 1(b), 1(c), and 1(d)). Albumin was characterized by high and narrow peaks in all stages of the laying period. After the onset of laying, a more marked postalbumin fraction was observable in all hens on the cathodal side of the albumin peak (Figure 1(b)), which in 38-week-old hens (period of maximum egg production) appeared only as a shadow after the main albumin fraction (Figure 1(c)). In week 60 of age, this shadow disappeared and only a typical peak of albumin was visible. The *α*-globulins migrated in *α*_1_- and *α*_2_-subfractions. The *α*_1_-globulin fraction was presented as a low distinct single peak in all stages of the evaluated laying period. Compared to *α*_1_-globulin zone, the *α*_2_-globulin fraction was characterized by a moderate and sharper peak, which in hens at the age of 38 weeks (at maximum egg production) showed a twin peak (Figure 1(c)). This pattern was observable also in hens at the age of 60 weeks (Figure 1(d)). The *β*-globulin fraction was characterized by higher and sharper electrophoretic amplitude than the *γ*-globulin fraction and after the onset of laying tended to show a twin peak. The *γ*-globulin fraction was not clearly distinguishable from the *β*-globulin fraction and showed a shoulder on the right side of the electrophoretogram.

Descriptive statistics of data for the relative concentrations of protein fractions during the pre- and postlaying periods are shown in Table 2. The relative values of most of the separated serum protein fractions were significantly affected by laying. In the relative concentrations of albumin, we observed a trend of gradually decreasing values till week 38, which represents the period of maximum egg production. From week 50, the values started to increase and dropped again at the end of the evaluated 1-year laying period. Compared with the prelaying period, higher relative concentrations of *α*_1_-globulins during the early and maximum laying period were observed, while at the end of the evaluated period, the values started to decrease. A significant increase in values in the early laying period was found in the relative concentrations of *α*_2_-globulins, reaching the maximum in week 38 (maximum egg production). In the following laying period, a significantly lower ratio of this protein fraction was recorded again. However, these values were still higher compared to those obtained in the pre- and early laying period. In the relative concentrations of *β*-globulins, a slight increase in means was observed till week 29 with a consecutive gradual decrease in values till week 60. The lowest mean value was recorded before the laying period and the significantly highest mean value at the end of the monitored period. The relative concentrations of *γ*-globulins decreased significantly after the beginning of laying (*p* < 0.05) with the lowest mean values in weeks 22 and 38 (maximal egg production). After this period, a repeated increase in values was observed, but they were lower compared to the prelaying period. The A/G ratios were higher before and at the beginning of the laying period, and the lowest values were obtained in weeks 38 and 70, in periods with the highest egg production. These variations in the A/G ratio were statistically significant (*p* < 0.05).

**Figure 2:**
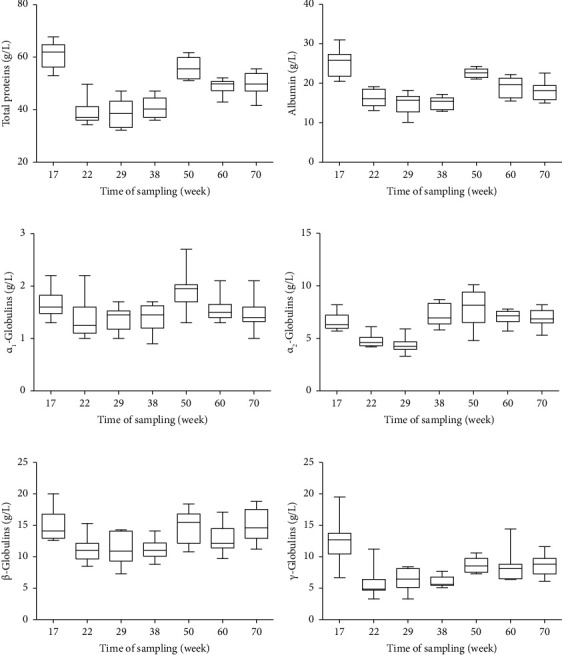
The distribution of the concentrations of total proteins (a), albumin (b), *α*_1_- (c), *α*_2_- (d), *β*- (e), and *γ*-globulins (f) in laying hens during the evaluated period. The plots show the median (line within the box), 25th, and 75th percentiles (box) and minimal and maximal values (whiskers).

The distribution of the concentrations of TP and individual protein fractions are presented in Figures 2(a), 2(b), 2(c), 2(d), 2(e), and 2(f). A significant effect of egg production was observed on the concentrations of total serum proteins (*p* < 0.001) and all the separated protein fractions (*p* < 0.001, Table 3). The highest mean total protein value was recorded in the prelaying period (Figure 2(a)). Compared with this value, a significant decrease was observed during the early laying period (*p* < 0.05), particularly between weeks 22 and 38. In week 50, a significant increase in mean total serum protein concentration was seen (*p* < 0.05), and TP values were also higher in the following period than in the first half of the laying period. Similarly, significant changes were observed in the absolute concentrations of albumin, with the highest mean value before laying and significantly lower values in the early and maximum laying period (Figure 2(b)). Significant fluctuations were observed in the absolute concentrations of *α*_1_- and *α*_2_-globulins (*p* < 0.001), with the highest mean values in week 50 (Figure 2(c), 2(d)). Compared to the prelaying sample collection, the absolute concentrations of *β*-globulins decreased significantly after the beginning of laying (*p* < 0.05), with the lowest mean values recorded in weeks 22 and 38 (maximum egg production) (Figure 2(e)). In the following period, a repeated increase in *β*-globulin concentrations was observed. The absolute concentrations of *γ*-globulins decreased significantly in weeks 22 to 38 after the beginning of laying and in the period of maximum egg production (*p* < 0.05, Figure 2(f)). The mean value obtained in this period was lower approximately two times compared to the prelaying period. An increase in mean values was observed from week 50 till the end of the evaluated period (week 70); however, these values were lower than in the period before the start of laying.

## 4. Discussion

The breeding progress over the past years was enormous and created laying hens with a high potential of egg production over a prolonged period, which continuously lay a big amount of eggs. High egg production places great demands on the organism of poultry and may be associated with several changes in the biochemical processes during the laying period. This is related not only to energy and mineral metabolism but also to the serum protein pattern and distribution of blood serum proteins. Numerous serum proteins are present in the blood of hens during the laying period, which have several physiological roles and are also an important production feature [3]. Many of them are the main components of egg yolk, and others serve as egg white precursors [16]. Our study showed in hens reared under free-range farming conditions marked changes in the concentrations of total serum proteins during the 1-year laying period. The most marked alterations were observed during the early laying period. Shortly after the onset of laying, particularly between weeks 22 and 38, a marked drop in values was found. This might reflect the initiation of egg laying and transfer of serum proteins from the circulation of hens to the yolk. After reaching the maximum egg production, a repeated increase in total protein values was observed. However, the concentrations of total serum proteins obtained in this period of laying were still lower than those measured before laying. Burnham et al. [12] recorded in commercial Single-Combed White Leghorn laying hens only minimal changes in the total serum protein concentrations during the laying period. In studies conducted by Suchý et al. [13] and Pavlík et al. [14] in Isa Brown hens reared under a cage housing system, a slight increase in TP values was recorded in the first half of the laying period, which was related to the increased proteosynthesis during the higher intensity of egg production. On the other hand, Hrabčáková et al. [17] showed in common pheasant hens housed in enhanced cages a significant drop of total protein values between weeks 6 and 12 of laying, when the rate of laying reached its maximum. This indicates that the period shortly after the initiation of laying is the most critical period for hens reared under free-range farming conditions. In this period, the intensive egg production and the transfer of serum proteins into the egg may markedly alter their concentrations in the blood. The differences observed in the dynamics of changes in total serum protein concentrations compared to other authors may be associated with differences in housing and rearing systems, breeds of hens, and enrichment of feed in commercial housing systems.

In hens included in our study, prealbumin fraction was not found as a distinct electrophoretic band. It was clearly observable only in 4 of 10 hens at the age of 29 weeks and was in minimal quantities. Previously, prealbumin was visualized in young chickens as a small band anodic to the albumin fraction [15]. On the other hand, Filipović et al. [3] did not observe the presence of prealbumin fraction in broiler chickens. The presence of clearly separated prealbumin fraction is more typical for some bird species, especially many psittacine species. A large prealbumin fraction is common in cockatiels, but it is either not present or only minimal in African grey parrots. On the other hand, budgies and quaker parakeets may have a larger amount of prealbumin than albumin [18]. Our study showed that hens housed in a cage-free system have very low or negligible prealbumin fraction, and it is not possible to clearly separate it from the albumin fraction. Alterations during the laying period have been observed in the concentrations of albumin. We recorded a more marked decrease in values after the onset of laying, which remained lower till week 38, when the laying capacity approached a maximum. Studies performed earlier showed that serum albumin concentrations in hens are not affected by egg laying [19, 20]. Gyenis et al. [21] described in Leghorn-type layers an increasing trend for the albumin values before egg production due to somatic growth and general anabolic processes and decreased after the beginning of egg production. It was suggested previously that of serum proteins albumin and predominantly gamma-globulins are transported from the circulation of the hen to the yolk [22]. Our results indicate that the laying cycle and production in hens reared under free-range farming conditions may also affect the concentrations of other globulin fractions. The relative percentages of *α*_1_- and *α*_2_-globulins increased slightly after the beginning of egg production. Their highest concentrations were obtained in periods when egg production approached the maximum. Several acute phase proteins migrate into these fractions, from which *α*_1_-acid glycoprotein, serum amyloid A, ceruloplasmin, and PIT54 (corresponding to haptoglobin in mammals) were in poultry identified as of great importance [23–26]. Since laying, especially the beginning of egg production, represents a considerable metabolic stress for hens, the concentrations of *α*-globulins may increase due to the processes associated with the formation and laying of eggs. However, the alterations in the distribution of globulin fractions during the laying cycle have not yet been completely understood. Studies have shown that several acute phase proteins (*α*_1_-acid glycoprotein, serum amyloid A, ceruloplasmin) not only may change in response to pathological processes but may also be influenced by physiological stress such as age and intensive growth in domestic birds [27–29]. Therefore, further studies are necessary to determine which specific serum proteins are responsible for the alterations observable in the serum protein pattern during the laying cycle.

In the *β*-globulin fraction, a significant decrease was found after the beginning of laying. The lowest values were found in the early laying period and in week 38 when the maximum egg production was achieved. Ovotransferrin is the most important protein from this fraction, which shows marked structural similarity with mammalian lactoferrin and transferrin [30, 31]. In laying hens, ovotransferrin is synthesized under the control of estrogen [32] and is a major constituent of egg white, as well as a matrix protein of the eggshell membranes [33]. Some specific glycophospholipoproteins, such as vitellogenin, are also present in the blood serum of laying hens. This serves as the primary egg yolk precursor protein and plays a role in the transport of circulating nondiffusible protein-bound calcium, which is important for eggshell formation [34, 35]. Therefore, the marked decrease in *β*-globulin concentrations after the beginning of egg production may reflect the transfer of these proteins from this fraction from the circulation and incorporation into the egg. Following laying, a significant decrease in total plasma protein, albumin, and *β*-globulin concentrations was found also in pigeons. This indicates the transfer and use of these proteins for yolk production [36]. On the other hand, increases in *α*_1_-, *α*_2_-, and *γ*-globulin fractions were recorded during this period. Seeing that there are scarce data about the changes in the aforementioned proteins throughout the production period of hens, further studies should be performed to describe their distribution in the blood serum of hens reared under open farm conditions, as well as commercial cage housing systems.

The primary components of the *γ*-globulin fraction are immunoglobulins IgΥ (equivalent to both IgE and IgG in mammals with similar immune response functions), IgM, and IgA [37]. According to Campbell and Dein [38] and Capitelli and Crosta [39], during egg production, estrogen-induced hyperproteinemia may occur due to the increased production of globulins to complete egg formation because most of the yolk proteins are globulins. In laying hens, the circulating immunoglobulins from the serum are transferred to the egg yolk [40, 41]. Therefore, the concentrations of immunoglobulins are reported to be markedly lower in hens after the onset of laying when compared to chickens [42]. In our study, a marked decrease in *γ*-globulin concentrations was found after the beginning of egg production, and the values were higher from week 50, after the period when the maximum egg production was reached. However, in this period, they no longer reached the values of the period before the start of laying. Seeing that immunoglobulins act as a critical part of the immune responses, the increase in gamma globulins may reflect also enhanced defense mechanisms and better immune status. The aforementioned changes in the protein fractions in different stages of the production period of laying hens also resulted in alterations in the A/G ratio. The lowest values were recorded in weeks 38 and 70, in the period with the highest egg production. As there are no data on the A/G ratio in the literature for laying hens, the presented results represent a significant addition to knowledge about this parameter within the protein profile of laying hens.

## 5. Conclusions

The results present and describe the serum protein electrophoretograms in hens reared under free-range farming conditions in relation to changes in the size and shape of five protein fractions during the 1 year laying period. Significant changes throughout the evaluated period were found in the concentrations of all the separated fractions. The most marked alterations were obtained after the beginning and in the early laying period. The concentrations of total serum proteins, albumin, *α*_2_-, *β*-, and *γ*-globulins decreased significantly compared to values recorded before laying and started to increase after reaching the maximum egg production. These findings suggest that the beginning of egg production is the most critical period in the laying cycle of hens reared under these farming conditions. In this period, the most important protein substances are transferred from the circulation of the hen to the egg, which thus serves as one of the most important natural sources of many nutrients, including high-quality proteins. Besides, the offspring develops outside the mother's body in eggs, and all nutrients needed for the embryo to fully develop must be provided in the egg before it is laid. Because of the housing system and composition of feed for laying hens, the beginning of egg production may be accompanied by various changes in the distribution of blood serum proteins, and further investigations are needed to describe these alterations during the laying period.

## Figures and Tables

**Table 1:** Chemical composition of the used laying hens' diets and additives in the diets.

Composition of feed in 1 kg of dry matter	Units	Type of feed mixture			
		GCH^1^	LCH^2^	LHC^3^	LHCo^4^
Crude protein	%	15.7	16.0	15.2	15.1
Crude fiber	%	3.75	5.0	4.7	3.2
Oil and fat	%	3.3	3.5	4.0	2.12
Ash	%	4.2	6.2	12.4	9.6
Lysine	%	0.66	0.72	0.7	0.76
Methionine	%	0.34	0.31	0.35	0.36
Ca	%	1.17	1.3	3.8	2.88
P	%	0.68	0.60	0.43	0.5
Na	%	0.16	0.17	0.16	0.18
Vitamin A	IU·kg^−1^	10,000	10,000	10,000	13,500
Vitamin D3	IU·kg^−1^	2600	2500	2300	3000
Vitamin E^5^	mg·kg^−1^	—	50.0	15.0	—
Fe^6^	mg·kg^−1^	200.0	40.0	50.0	250.0
Cu^7^	mg·kg^−1^	16.0	15.0	15.0	25.0
Mn^8^	mg·kg^−1^	130.0	60.0	70.0	126.0
Zn^9^	mg·kg^−1^	110.0	70.0	55.0	85.0
Se^10^	mg·kg^−1^	0.3	0.3	0.4	—
I^11^	mg·kg^−1^	1.0	1.0	1.0	—

^1^GCH—feed for growing chickens.

^2^LCH—feed for chickens intended for laying.

^3^LHC—feed for laying hens classic.

^4^LHCo—feed for commercial laying hens.

^5^Vitamin E as alfa tocopherol acetate.

^6^Fe as ferrous sulfate monohydrate.

^7^Cu as copper sulfate pentahydrate.

^8^Mn as manganese oxide.

^9^Zn as zinc oxide (GCH) or zinc sulfate monohydrate.

^10^Se as sodium selenite.

^11^I as calcium iodate anhydrous.

**Table 2:** Changes in the relative concentrations of serum protein fractions (%) and albumin/globulin ratio (A/G) in hens during the observed laying period.

Parameter		Time of sampling							*p* value
		0	1	2	3	4	5	6	
Albumin	*x*	41.6	41.6	39.2	36.7	40.5	38.7	36.0	0.0283
	sd	4.7	5.8	6.0	2.0	2.5	4.2	4.2	
	Median	41.4	41.1	38.5	36.2	40.2	39.8	36.5	

*α*_1_-Globulins	*x*	2.8	3.5	3.6	3.5	3.4	3.2	2.9	0.0070
	sd	0.5	0.6	0.6	0.5	0.6	0.4	0.7	
	Median	2.8	3.4	3.5	3.6	3.4	3.2	2.7	

*α*_2_-Globulins	*x*	10.8^a^	12.2^a^	11.3^a,b^	17.7^c^	14.1^d^	14.4^d^	13.9^d^	< 0.0001
	sd	1.2	0.9	1.3	2.2	2.7	1.3	1.5	
	Median	10.9	12.3	11.4	17.3	14.6	14.3	14.0	

*β*-Globulins	*x*	24.5^a^	28.4	29.4	27.5	26.5	26.5	29.8^b^	0.0147
	sd	2.7	3.1	3.9	2.5	3.1	5.0	3.7	
	Median	27.4	29.2	29.4	27.9	27.1	24.9	29.8	

*γ*-Globulins	*x*	20.4^a^	14.4^b^	16.3	14.7	15.5	17.3	17.4	0.0301
	sd	5.6	3.8	3.2	1.4	1.9	4.9	2.4	
	Median	20.1	13.4	17.2	14.6	15.4	16.3	17.3	

A/G	*x*	0.72	0.73	0.67	0.58	0.68	0.64	0.57	0.0319
	sd	0.14	0.18	0.18	0.05	0.07	0.11	0.10	
	Median	0.71	0.70	0.63	0.57	0.67	0.66	0.57	

*Note:p* value—significance of the ANOVA test; *x*—mean value.

Abbreviation: sd, standard deviation.

^a,b,c,d^Statistically significant difference between the sample collections (level of significance *p* < 0.05).

**Table 3:** Changes in the absolute concentrations of total serum protein (TP) and protein fractions (g/L) in hens during the observed laying period.

Parameter		Time of sampling							*p* value
		0	1	2	3	4	5	6	
TP	*x*	60.8^a^	39.1^b^	38.5^b^	40.8^b^	55.9^a,c^	48.9	50.0^a,c^	< 0.0001
	sd	5.0	5.2	5.3	3.9	4.3	2.7	4.3	
	Median	61.9	37.1	38.6	40.3	55.5	49.8	49.8	

Albumin	*x*	25.3^a^	16.2^b^	15.0^b^	14.9^b^	22.6^c^	19.0^d^	18.0^b,d^	< 0.0001
	sd	3.4	2.2	2.4	1.6	1.2	2.6	2.4	
	Median	25.9	16.1	15.7	15.4	22.7	19.6	18.2	

*α*_1_-Globulins	*x*	1.7	1.4^a^	1.4^a^	1.4^a^	1.9^b^	1.6	1.5^a^	0.0005
	sd	0.3	0.4	0.2	0.3	0.4	0.2	0.3	
	Median	1.6	1.3	1.5	1.5	1.9	1.5	1.4	

*α*_2_-Globulins	*x*	6.6^a^	4.8^b^	4.3^b,c^	7.2^a,d^	7.9^d^	7.0^a,d^	6.9^a,d^	< 0.0001
	sd	0.8	0.6	0.7	1.0	1.8	0.7	0.8	
	Median	6.3	4.6	4.3	7.0	8.2	7.2	6.9	

*β*-Globulins	*x*	14.9^a^	11.2^b^	11.4^b,c^	11.2^b,d^	14.9^a,c^	13.0	14.9^e^	< 0.0001
	sd	2.4	2.1	2.4	1.6	2.5	2.4	2.5	
	Median	14.1	11.0	10.9	11.0	15.5	12.2	14.6	

*γ*-Globulins	*x*	12.4^a^	5.7^b^	6.3^b,c^	6.0^b,c^	8.7^a,c^	8.5	8.7^a,c^	< 0.0001
	sd	3.6	2.2	1.6	0.8	1.1	2.3	1.6	
	Median	12.7	4.9	6.5	5.6	8.6	8.1	8.9	

*Note:p* value—significance of the ANOVA test; *x*—mean value.

Abbreviations: sd, standard deviation; TPs, total proteins.

^a,b,c,d,e^Statistically significant difference between the sample collections (level of significance *p* < 0.05).

## Data Availability

All data pertaining to the current study are available from the corresponding authors upon a reasonable request.
